# B-cell receptor-guided delivery of peptide-siRNA complex for B-cell lymphoma therapy

**DOI:** 10.1186/s12935-015-0202-4

**Published:** 2015-05-07

**Authors:** Nunzia Migliaccio, Camillo Palmieri, Immacolata Ruggiero, Giuseppe Fiume, Nicola M Martucci, Iris Scala, Ileana Quinto, Giuseppe Scala, Annalisa Lamberti, Paolo Arcari

**Affiliations:** Department of Molecular Medicine and Medical Biotechnology, University of Naples Federico II, Via S. Pansini 5, I-80131 Naples, Italy; Department of Experimental and Clinical Medicine, University of Magna Graecia, Viale Europa, I-88100 Germaneto, Catanzaro, Italy; Department of Molecular Medicine and Medical Biotechnology, University of Naples Federico II, Via S. Pansini, I-80131 Naples, Italy

**Keywords:** Id-peptide, Peptide-siRNA complex, Peptide engineering, B lymphoma cells, Minimal residual disease (MRD)

## Abstract

**Background:**

Despite the clinical response of conventional anticancer therapy, including chemotherapeutic treatments, radiation therapy and corticosteroids, tumorigenic B-cell lymphomas show an incomplete response to clinical practices that result in a minimal residual disease (MRD) where few residual neoplastic cells undetected *in vivo*, replenish the cancer cell reservoir. This scenario, which is also shared with other cancer diseases, requires the development of strategies to advance in novel, selective targeting toward the tumorigenic cells that survive to the anticancer agents.

**Methods:**

Here, we have taken advantage of the therapeutic properties of an idiotype specific peptide (pA20-36) that bind specifically to murine B-lymphoma cells in the setting of an anti cancer strategy, based on the selected delivery of electrostatic-based complex, peptide-siRNA. To this end, two engineered, arginine rich, peptides that included the pA20-36 targeting sequence were designed to bind fluorescent-labelled siRNA. One peptide presented 9 Arg at the C-terminal of pA20-36 whereas the other included 5 Arg at the N- and C-terminus, respectively.

**Results:**

Compared to the control and random peptide-siRNA complexes, both pA20-36-siRNA complexes were endowed with the selective delivering of fluorescent-labelled siRNA toward the A20 murine B-cell lymphoma, as evaluated by cytofluorimetry and confocal microscopy, whereas fluorescent-labelled siRNA alone was not internalized in the selected cells. Compared to peptide controls, the use of the modified pA20-36 peptides complexed with siRNA anti-GAPDH and anti-Bcl2 showed a down-regulation in the expression levels of the corresponding genes.

**Conclusions:**

Peptide-siRNA complex can be suitable tool for both selective peptide-driven cell targeting and gene silencing. In this setting, the improvement of this strategy is expected to provide a safe and non-invasive approach for the delivery of therapeutic molecules.

## Background

The targeted delivery of siRNA has recently emerged as a powerful therapeutic tool in different diseases, including cancer [1]. A major requirement for the targeted delivery of therapeutic siRNA in cancer concerns the specificity for target cells, meaning that the delivery systems must be highly specific for the tumor cells and not be able to exhibit the toxic effects of its cargo in normal cells. This issue may be fulfilled by targeting ligands for cell receptors to delivery siRNA to specific cell and/or tissue types. Commonly used targeting ligands include aptamers, cell penetrating peptides, antibodies, peptides or proteins, and small molecule ligands [2-5].

Peptides are the most promising tools for siRNA delivery, as they may fulfill different major requirements for an efficient siRNA delivery, including siRNA attachment, receptor recognition, disruption of endosomal membrane and intracellular trafficking [6-8]. Up to now a number of peptide ligands has been used to successfully deliver siRNA into target cells, with improvement of bio-available and specific delivery of nucleic acids to target cells [9].

The idiotype determinants of the immunoglobulin B-cell Receptor (Ig-BCR) of B cell malignancies are unique for a given clonal population and function as specific tumor antigen that may be exploited for targeted therapies. In the last years, we identified idiotype-specific peptides for the Ig-BCR of B cell tumors, named “Id-peptides”, that showed the following properties: a) binding to tumor target cells with high specificity and sensitivity, both *in vitro* and *in vivo*; b) internalization into target tumor cells by a BCR-mediated endocytosis; c) specific delivering of a cargo protein into target tumor cells [10,11]. Therefore, we used the specific identified peptide endowed with high-affinity toward highly aggressive murine A20 lymphoma cells [12-14]. Here we addressed the relevance of Id-peptides as carriers for the specific delivery of siRNA to target neoplastic B cells.

## Results

### Peptide-siRNA complexes cellular uptake

The Id-peptide pA20-36 is a specific binder for the surface Ig-BCR of A20 B-cell lymphoma cells. With the aim of evaluating the Id-peptide for the specific delivery of siRNA to target neoplastic B cells, we designed two new peptides, each composed of two modules: a N-terminal region corresponding to the original pA20-36 sequence targeting the Ig-BCR of the A20 B cells and a C-terminal or C- and N-terminal stretch of basic aminoacids (Arginine) that promotes the formation of electrostatic based siRNA-peptide complexes [15]. The 5R-A20-36-5R peptide contained two additional stretches of 5 arginine residues flanking the pA20-36 sequence whereas the pA20-36-9R contained an additional stretches of 9 arginine residues at the C-terminal region of the pA20-36 sequence. Both constructs contained D-Arg at the extremity and the substitution of L-arginine for the enantiomeric D-arginine was made for protease protection [16].

**Figure 1:**
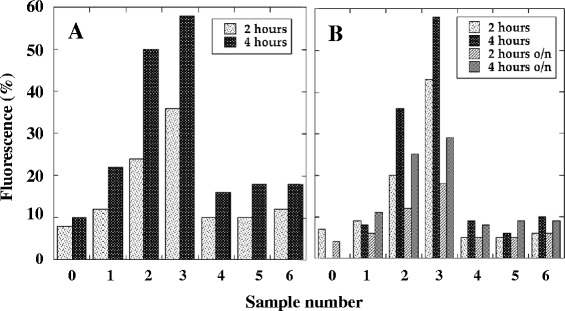
Fluorescence analysis of A20 cells after treatment with peptide-siRNA complexes. Peptide-siRNA complexes were incubated with A20 cells for the indicated times and analyzed at cytofluorimeter after 3 washes. **A)** and **B)** Permealized A20 cells. Samples: 0, untreated control cells; 1, fluorescent siGLORNAi alone; 2, A20-36-9R-siGLORNAi; 3, 5R-A20-36-5R-siGLORNAi; 4, RND-9R-siGLORNAi; 5, 5R-RND-5R-siGLORNAi; 6, A20-36-siGLORNAi.

These peptides were complexed with fluorescent-labelled control siGLORNAi and tested for their ability to drive the selective internalization of the siRNA in A20 cells. As reported in Figure 1, the cytofluorimetric evaluation of the red fluorescence indicated that compared to untreated cells (Figure 1A and B, sample 0) or to cells treated at zero time, that showed fluorescent values similar to those of untreated samples (not shown), both peptide-siRNA complexes were able to delivery siRNA in A20 cells with respect to the corresponding control peptide-siRNA complexes. In particular, with respect to A20-36-9R-siGLORNAi, 5R-A20-36-5R-siGLORNAi appeared to be more efficient. This result was obtained by the cytofluorimetric analysis of permeabilized A20 cells from two independent experiments (Figure 1A and B, samples 2 and 3). The same effect was not observed with the control random peptides and with the non-modified A20-36-siRNA (Figure 1A and B, samples 4, 5 and 6).

### Confocal microscopy of peptide-siRNA complexes cellular uptake

**Figure 2:**
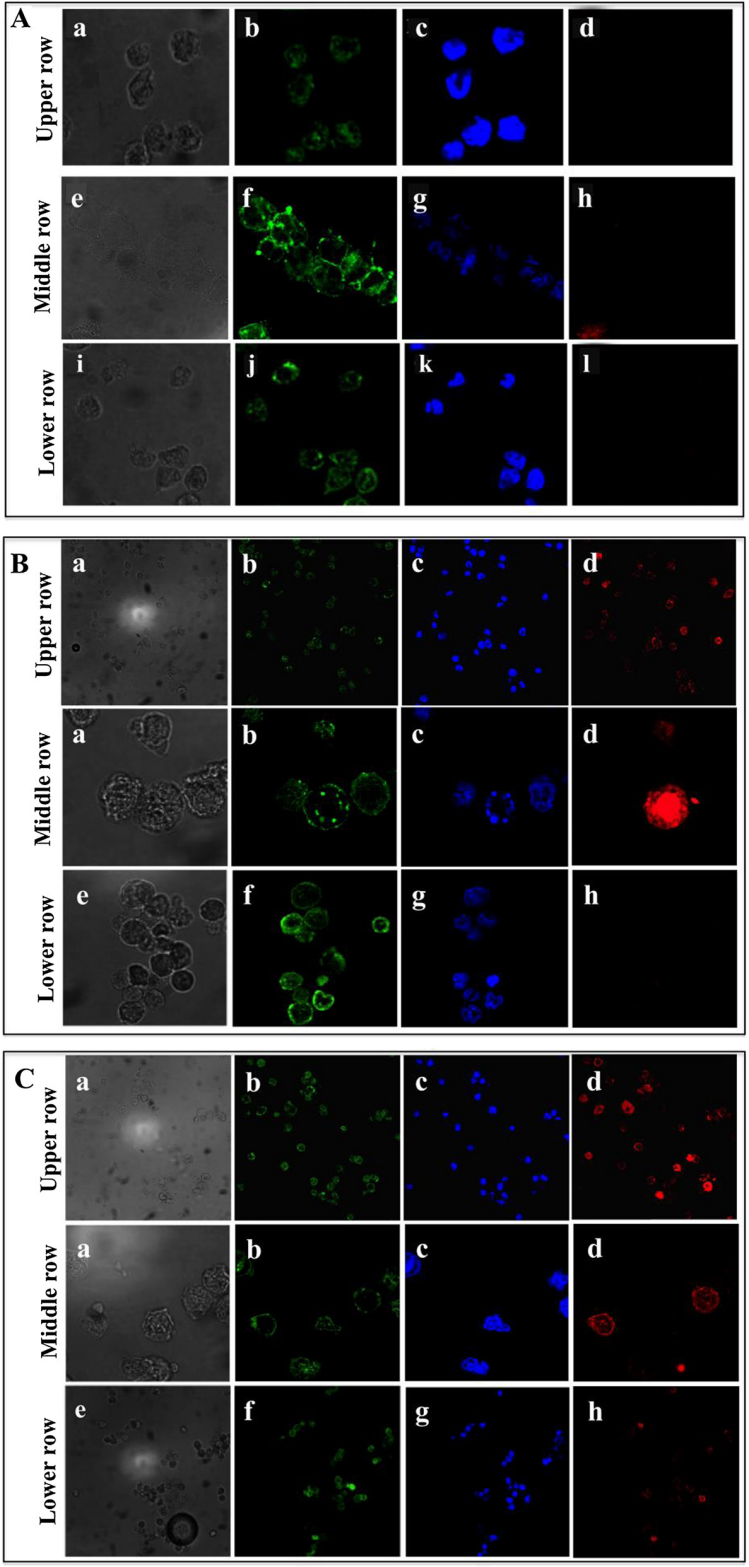
Confocal microscopy of A20 cells after treatment with labeled siGLORNAi and peptide-siGLORNAi. A20 cells were incubated with siGLORNAi for 4 hours. After 3 washes, cells were analyzed at confocal microscopy. **(A)**. Upper row: untreated A20 cells. Middle row: A20 cells after treatment with siGLORNAi alone. Lower row: A20 cells transfected with siGLORNAi by using Lipofectamine 2000. Peptide-siGLORNAi complexes were incubated with A20 cells for 4 hours and after 3 washes, cells were analysed at confocal microscopy. **(B)**. Upper row and Middle row: images at low and high magnification, respectively of A20 cells treated with A20-36-9R-siGLORNAi. Lower row: A20 cells treated with RND-9R-siGLORNAi. **(C)** Upper row and Middle row: images at low and high magnification, respectively of A20 cells treated with 5R-A20-36-5R-siGLORNAi. Lower row: A20 cells treated with 5R-RND-5R-siGLORNAi. a, e and i) Light microscopy of A20 cells; b, f and j) Membrane staining; c, g and k) Nuclear staining; d, h and l) fluorescence of siGLORNAi.

To highlight the A20 BCR-mediated internalization of the peptide-siRNA, we analyzed the localization of various complexes using confocal microscopy. As reported in Figure 2, compared to untreated A20 cells (Figure 2A, upper row), fluorescent siGLORNAi alone was not able to enter A20 cells since no fluorescence at 570 nm was detectable (Figure 2A, middle row, negative control). Moreover, no fluorescence at 570 nm was detectable when A20 cells were transfected with siGLORNAi by using Lipofectamine (Figure 2A, lower row). When the cells were treated with siGLORNAi complexed with A20-36-9R or 5R-A20-36-5R peptides, a red fluorescent signal was instead detected (Figure 2B and C, upper and middle rows) whereas treatment of the A20 cells with siGLORNAi complexed with the random peptides (RND-9R and 5R-RND-5R) did not show any siRNA internalization (Figure 2B and C, lower rows).

### Effect of siRNAGAPDH-peptide and siRNABcl2-peptide complexes on gene silencing

**Figure 3:**
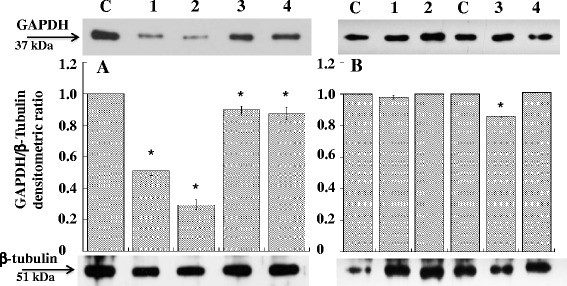
Down-regulation of GAPDH expression in NIH/3T3 and A20 cells. siRNAGAPDH was transfected with Lipofectamine 2000 at 37°C for 30 and 72 hours in (A) NIH/3T3 and (B) A20 cells. After incubation, cells extracts were analysed by western blot with anti-GAPDH antibody. (A) Lanes: C, untreated cells; 1 and 2 cells treated for 30 hours with 100 and 250 pmoles of siRNAGAPDH, respectively; 3 and 4, cells treated for 72 hours with 100 and 250 pmoles of siRNAGAPDH, respectively. (B) Lanes: C, untreated cells; 1 and 2 cells treated for 30 hours with 100 pmoles of siRNAGAPDH and siRNASCR, respectively; 3 and 4, cells treated for 72 hours with 100 pmoles of siRNAGAPDH and siRNASCR, respectively. Data from three experiments are reported as the mean ± SD. * p < 0.05 compared to control.

**Figure 4:**
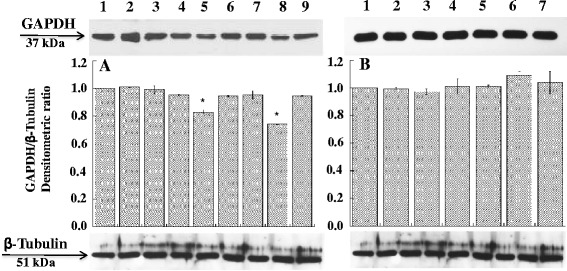
Effect of siRNAGAPDH on the expression levels of GAPDH. A20 cells were treated with siRNAGAPDH and peptide-siRNAGAPDH complexes for 48 hours. After incubation cell extracts were analyzed by western blot with anti-GAPDH antibody. **A**. Lanes: 1, control; 2, siRNAGAPDH; 3, siRNASCR; 4, A20-36-9R; 5, A20-36-9R-siRNAGAPDH; 6, A20-36-9R-siRNASCR; 7, 5R-A20-36-5R; 8, 5R-A20-36-5R-siRNAGAPDH; 9, 5R-A20-36-5R-siRNASCR. **B**. Lanes: 1, Control; 2, RND-9R; 3, RND-9R-siRNAGAPDH; 4, RND-9R-siRNASCR; 5, 5R-RND-5R; 6, 5R-RND-5R-siRNAGAPDH; 7, 5R-RND-5R-siRNASCR. Data are from three experiments are reported as the mean ± SD. * p < 0.05 compared to control and to all other samples.

**Figure 5:**
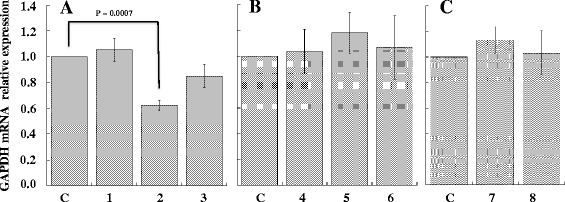
Effect of siRNAGAPDH on the expression levels of GAPDH mRNA. 5 × 10^5^ A20 cells were treated with A20-36-9R peptide in absence (control) or presence of siRNAGAPDH. 48 hours post-treatment, total RNA was extracted and analysed by qRT-PCR to evaluate the GAPDH mRNA expression relative to β-actin. **A**. Lanes: C, control; 1, A20-36-9R; 2, A20-36-9R-siRNAGAPDH; 3, A20-36-9R-siRNASCR. **B**. Lanes: C, control; 4, RND-9R; 5, RND-9R-siRNAGAPDH; 6, RND-9R-siRNASCR. **C**. Lanes: C, control; 7, siRNAGAPDH; 8, siRNASCR. Values of mean ± SD (n = 3) are shown.

**Figure 6:**
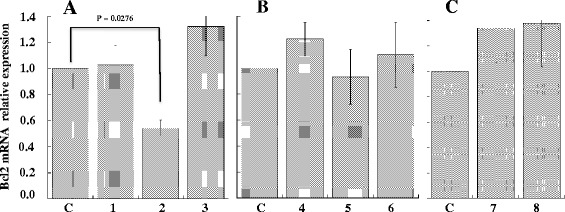
Effect of siRNABcl2 on the expression levels of Bcl2 mRNA. 5 × 10^5^ A20 cells were treated with 5R-A20-36-5R peptide in absence (control) or presence of siRNABcl2. 48 hours post-treatment, total RNA was extracted and analysed by qRT-PCR to evaluate the Bcl2 mRNA expression relative to β-actin. **A**. Lanes: C, control; 1, 5R-A20-36-5R; 2, 5R-A20-36-5R-siRNABcl2; 3, 5R-A20-36-5R-siRNASCR. **B**. Lanes: C, control; 4, 5R-RND-5R; 5, 5R-RND-5R -siRNABcl2; 6, 5R-RND-5R-siRNASCR. **C**. Lanes: C, control; 7, siRNABcl2; 8, siRNASCR. Values of mean ± SD (n = 3) are shown.

In order to evaluate the capability of the complex to decrease the levels of GAPDH mRNA, we used the same peptides complexed to siRNA directed against GAPDH mRNA (siRNAGAPDH). As controls, we first analysed the efficiency of siRNA anti-GAPDH to silence GAPDH expression in NIH/3T3 and in A20 cells by using lipofectamine (Figure 3). Compared to basal expression of GAPDH, siRNAGAPDH was able to downregulate in NIH/3 T3 cells the level of the enzyme of about 40% after 30 hours from transfection using both 100 or 250 pmoles of siRNAGAPDH whereas in A20 cells, GAPDH silencing was instead of about 10% after 72 hours from transfection using 100 pmoles of siRNAGAPDH. Treatment of A20 cells with peptide-siRNAGAPDH complex resulted instead relatively more efficient compared to lipofectamine (Figure 4). In fact, the A20-36-9R-siRNAGAPDH and 5R-A20-36-5R-siRNAGAPDH complexes were able to decrease the expression levels of GAPDH after 48 hours of about 15% and 25% lower respectively, thus confirming what observed by cytofluorimetry. To ascertain if the observed reduction occurred also at transcription level, qRT-PCR was settled on RNA samples prepared from A20 cells after their treatment with A20-36-9R-siRNAGAPDH in the same experimental conditions. As reported in Figure 5, compared to β-actin as control, a reduction of about 38% of GAPDH mRNA was detected. To confirm this behaviour, we then applied this strategy to silence the anti-apoptotic expression of Bcl2, highly expressed in lymphoma cells [17]. As shown in Figure 6, compared to controls, treatment of A20 cells with 5R-A20-36-5R-siRNABcl2 resulted in a reduction of Bcl2 mRNA of about 45%.

## Discussion

**Figure 7:**
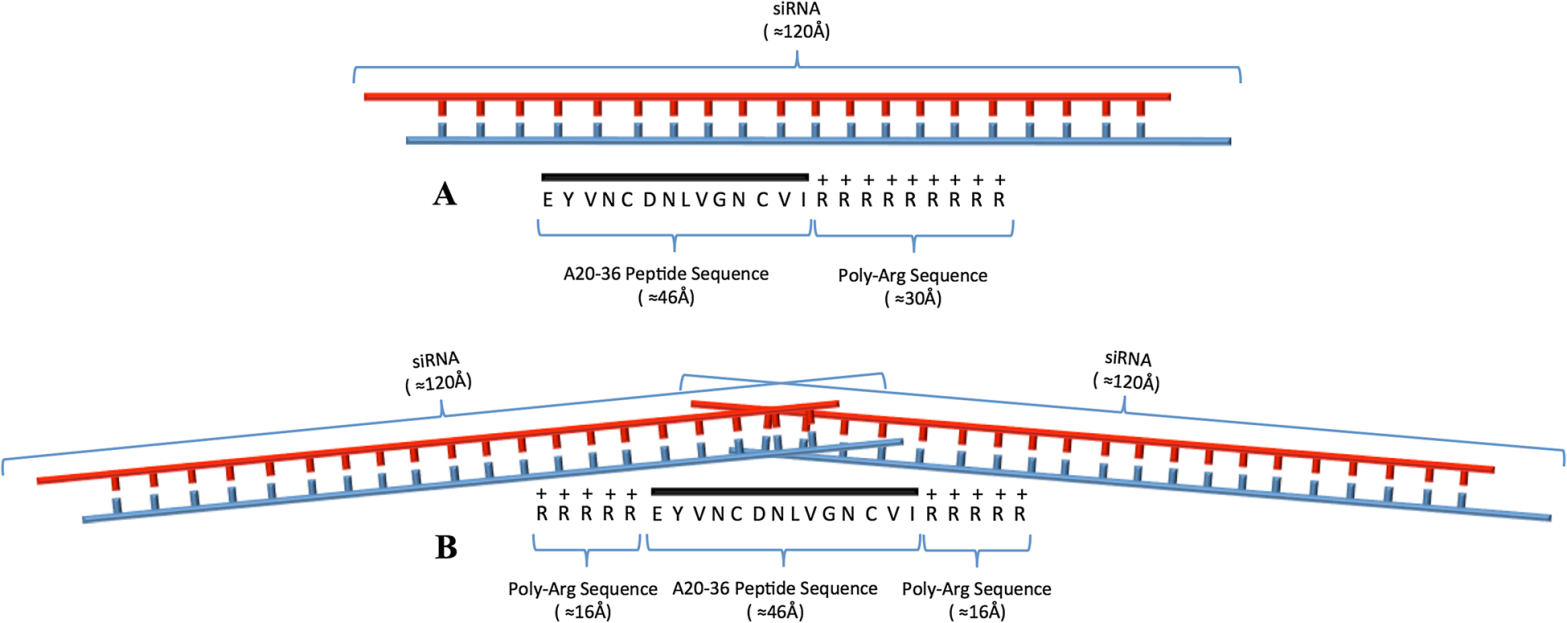
Peptide-siRNA complexes. Graphical representation of the complexes between A20-36-9R-siRNA **(A)** and 5R-A20-36-5R-siRNA **(B)**. The approximate canonical secondary structure distances are reported in parenthesis.

In this work we report a non-covalent peptide-based strategy for the delivery of molecules into cultured cancer cells using the capability of peptide to form stable electrostatic complex with siRNA. We have taken advantage from a previous work were we have reported the targeting specificity and the therapeutic properties of an idiotype-specific peptide toward a murine B lymphoma engrafted in syngenic, immune competent mice [11]. In the mentioned work it was showed that IA20-36 Id-peptide was specifically internalized into target cells with a mechanism of receptor-mediated endocytosis. The A20-36 Id-peptide triggered a BCR-mediated signaling that induced apoptosis of the target cells. Two different engineered peptides containing the sequence of the A20-36 Id-peptide recognized by BCR were designed. One contained five additional arginines at both N- and C-terminus of the Id-peptide sequence the other contained nine arginines only at the C-terminus. These peptides, besides containing the sequence for selective B cells recognition, were also able to make stable electrostatic complex with negatively charged siRNA [18,19] (Figure 7). Using a commercially available fluorescent control siRNA these complexes were able to selectively recognize the murine A20 cells for siRNA delivery as evaluated by cytofluorimetric measurements at 570 nm. The additional confocal microscopy also highlighted the internalization of siRNA and this was specifically driven by A20-36 peptide since no internalization was observed using random peptides.

The selectivity of the siRNA peptide complexes relies on the ability of the pA20-36 peptide to target only the idiotypic determinants of the immunoglobulin receptor displayed on A20 B cells, and not of other immunoglobulin receptors or different cell surface receptors. In fact, pA20-36 peptide does not bind to the entire normal mouse PBMC and bone marrow cells, as well other B cell tumor cell lines (MC3, Raij, 5T33MM) and T cell lines (HPB-ALL, Jurkat) [11]. In fact, as ligand of the idiotypic determinants of the immunoglobulin B-cell Receptor (IgBCR), the specificity of each Id-peptide is strongly restricted to B cell clone (normal or neoplastic) that harbour the cognate IgBCR.

For potential delivery *in vitro*, we then examined if these peptides were able to delivery siRNAGAPDH by evaluating the expression levels of the corresponding protein in A20 cells. Our results suggest that the both A20-36-9R and 5R-A20-36-5R peptides were able to deliver siRNAGAPDH to B cells and down-regulate the protein expression. To confirm the effective siRNA-mediated gene down-regulation, we also evaluated by qRT-PCR in two separate experiments the mRNA level of GAPDH and Bcl2 using A20-36-9R and 5R-A20-36-5R in complex with siRNAGAPDH and siRNABcl2, respectively. Interestingly, the result obtained using 5R-A20-36-5R-siRNABcl2 was more promising compared to that of A20-36-9R-siRNAGAPDH and was in agreement with the finding that 5R-A20-36-5R peptide was more efficient than A20-36-9R (Figure 1). The siRNA internalization strategy here reported should have the great advantage of being based on the specific ligand-receptor interaction. There are several aspects of this approach that can be modified in order to improve its efficacy. Because the lower efficiency of A20-36-9R- and 5R-A20-36-5R-bound to siRNAs was most likely indicative of a lower protection against lysosome degradation, the use of chemically stabilized siRNA may enhance the efficacy on genes down-regulation. Encapsulation of stabilized siRNA entrapped into nanoparticle could greatly enhance half-life and bioavailability [20,21]. Liposomal and polymeric nanoparticles coated with targeting ligands represent one possibility since they have been already employed for delivery [22,23].

## Conclusions

In this work, we have exploited a novel method based on the chemistry of siRNA for the construction of stabilized siRNA-peptide complexes for the selective peptide-driven cell targeting and as appropriate tool for effective gene silencing. However, a major limitation of the Id-peptide approach for the specific delivery of siRNA (or other cargos) is the requirement of an Id-peptide selection procedure for each individual B-cell clone (patient). Nevertheless, this concern does not impair the main significance of the present work, which is the proof-of-concept of the specific delivery of a siRNA cargo into target cells accomplished by an Id-peptide.

## Materials and methods

### Materials

siGLO RISC-Free Control siRNA (siGLORNAi), chemically modified to impair processing and uptake by RISC, one-target plus SMART pool siRNAGAPDH and one-target plus SMART pool siRNABcl2 were from Thermo Scientific Dharmacon (GE Healthcare, CO, USA). Peptide synthesis was perfomed by CASLO Laboratory, Lyngby, Denmark. Hoechst Stains and Lipofectamine 2000 were from Invitrogen (CA, USA). Cholera Toxin B Subunit FITC Conjugate was from Sigma (MO, USA).

### Cell cultures

A20 is a murine cell line derived from a spontaneously arising tumor in an aged BALB/c mouse. It pathologically mimics the characteristics of human diffuse large B cell lymphoma [12]. Cell line was kindly provided by Prof. C. Palmieri (University of Catanzaro, Italy). The cell line was grown in suspension culture with RPMI 1640 medium (GIBCO, CA, USA), supplemented with 10% fetal bovine serum (GIBCO), 50 units/ml penicillin, 50 μg/ml streptomycin and 2 mM L-glutamine at 37 °C in a 5% CO_2_ atmosphere. NIH/3T3 is a murine embryonic fibroblast cell line. The cell line was provided by CEINGE, Advanced Biotechnologies (Naples, Italy). The cell line was grown in adhesion culture with Dulbecco's Modified Eagle's Medium (GIBCO, CA, USA), supplemented with 10% fetal bovine serum (GIBCO), 50 units/ml penicillin, 50 μg/ml streptomycin and 2 mM L-glutamine at 37°C in a 5% CO_2_ atmosphere.

### Cytofluorimetric analysis and confocal microscopy

Target peptides and random peptides (2 μl, 1 mg/ml, 11.58 μM) plus siGLORNAi (4 μl, 25 μM, 100 pmoles) were incubated at room temperature for 15 minutes at a 1:10 molar ratio in RPMI without FCS and then added to 50 μl of A20 cells at 20 × 10^6^/ml in RPMI 20% FCS. Samples were then incubated at 37°C for 2 hours and 4 hours. After 3 washes in 1× Phosphate Buffered Saline (PBS) (Invitrogen, CA, USA), cells were divided in two aliquots. One aliquot (10^7^/ml) was analysed by flow cytometry whereas the other aliquot was suspended in 2 ml of RPMI and incubated in 6 wells plates overnight and then analysed by flow cytometry. Cells before the cytofluorimetric analysis were permeabilized according to the following procedure: cells were resuspended in 100 μl of 1% paraformaldehyde and incubated at room temperature for 1 hour, washed twice with 200 μl 1× PBS, resuspended in 200 μl 1× PBS, 0.1% Triton X-100 (Sigma, MO, USA), 0.1% sodium cytrate and analyzed at flow cytometry.

The following A20 target-specific peptides were designed based on the peptide sequence reported by Palmieri et al., 2010 [11]. 5R-A20-36-5R, (D-Arg)(L-Arg)_4_-EYVNCDNLVGNCVI-(L-Arg)_4_(D-Arg); A20-36-9R; EYVNCDNLVGNCVI-(L-Arg)_8_(D–Arg); whereas as controls the following random peptides were designed: 5R-RND-5R, (D-Arg)(L-Arg)_4_-SSAYGSCKGPCSSGVHSI-(L-Arg)_4_(D-Arg); RND-9R, SSAYGSCKGPCSSGVHSI(L-Arg)_8_(D-Arg).

For confocal analysis, after incubation of the cells with complexes peptides-siGLORNAi for 4 hours as above reported, about 10,000 cells were plated on small glass disks in a 24 well dishes and let grown for 12 hours. At different times, disks were removed from the wells, washed three times with 1× PBS, stained with membrane stain Cholera Toxin B Subunit FITC Conjugate whereas cell nuclei were stained with Hoechst 33342 and subsequently observed with a Nikon Confocal Microscope C1 equipped with an EZ-C1 Software for data acquisition. Fluorescent image were collected, upon excitation according to dye manufacturing instructions.

### siRNAGAPDH and peptide-siRNAGAPDH complex

siRNAGAPDH was used for GAPDH mRNA down-regulation in NIH/3T3. 100 or 250 pmoles of siRNAGAPDH were transfected in the presence of Lipofectamine 2000 (Invitrogen, USA) according to manufacturing protocol. Samples were then incubated at 37°C for 30 and 72 hours. After incubation, cell extracts were then analysed by western blot with anti-GAPDH antibody.

A20-36 target peptides and random peptides plus siRNAGAPDH were incubated according the procedure above reported with A20 cells at 20 × 10^6^/ml in RPMI 20% FCS. After incubation, cells were suspended in 2 ml of RPMI and incubated in 6 wells plates for 48 and 72 hours. Cell extracts were then analysed by western blot with anti-GAPDH antibody.

### Western blotting analysis

Cells were collected, washed twice in 1× PBS and resuspended in 20–40 μl of lysis buffer (50 mM Tris–HCl pH 7.4, 1% NP40, 0,25% sodium deoxycholate, 150 mM NaCl, 1 μg/ml aprotinin, leupeptin, pepstatin, 1 mM Na_3_VO_4_, 1 mM NaF) for 30 min on ice and centrifuged at 14,000 × *g* for 20 min at 4°C. Protein concentration was determined according to Lowry et al., 1951 [24].

Proteins were separated by SDS-PAGE, electro-transferred to PVDF membrane, and incubated over-night at 4 °C with GAPDH monoclonal antibody at dilution of 1:2000. Anti-mouse secondary antibody conjugated with horseradish peroxidase (HRP) (Sigma) was used at a dilution of 1:20,000. The specific protein was visualized by an enhanced chemiluminescence detection reagent (SuperSignal West Pico, Molecular Probes, CA, USA), and exposed to X-ray film. All films were analysed by using Image J software 1.46r.

### qRT PCR

Total RNA extraction and qRT-PCR were performed as previously described [25]. Briefly, RNA aliquots (500 ng) were reverse transcribed using Random Examers and Superscript III Reverse Transcriptase (Invitrogen), according to the manufacturer's protocol. qRT-PCR was carried out with the iCycler iQ Real-Time detection system (Bio-Rad Laboratories) under the following conditions: 95°C, 1 min; (94°C, 10s; 60°C, 30s) × 40. Primers were as follows: β-actin, forward 5’-GGCACCACACCTTCTACAATGAG-3’ and reverse 5’-GGAGTCCATCACAATGCCTGTGG-3’; GAPDH, forward 5’-GTCAAGGCCGAGAATGGGAAGC-3’ and reverse 5’-AGAAGGGGCGGAGATGATGACC-3’; Bcl2, forward 5’-TCGCAGAGATGTCCAGTCAGC, reverse 5’-CCGGTTCAGGTACTCAGTCATC. GAPDH and Bcl2 expression levels were calculated relative to β-actin mRNA levels as endogenous control by using the formula 2^(Ct sample − Ct β-actin)^ [25].

### Statistical analysis

Data are reported as average and standard deviation. The statistical significance of differences among groups was evaluated with ANOVA, with the Bonferroni correction as post hoc test, using the software KaleidaGraph v. 4.1. The significance was accepted at the level of p < 0.05.

## Abbreviations

FITC: Fluorescein isothiocyanate
GAPDH: Glyceraldehyde-3-phosphate dehydrogenase
MRD: Minimal residual disease
PBS: Phosphate buffer saline
PVDF: Polyvinyl difluoride
RND: Random
SCR: Scramble
siRNA: Small interfering RNA
